# Maternal Intrusive Thoughts and Dissociative Experiences in the Context of Early Caregiving Under Varying Levels of Societal Stress

**DOI:** 10.3390/bs15060717

**Published:** 2025-05-23

**Authors:** Miriam Chasson, Jessica L. Borelli, Dana Shai, Orit Taubman – Ben-Ari

**Affiliations:** 1 Department of Psychiatry, Cambridge Hospital, Harvard Medical School, Cambridge, MA 02139, USA; 2 Department of Psychological Science, University of California, Irvine, CA 92697, USA; jessica.borelli@uci.edu; 3 School of Behavioral Sciences, The Academic College of Tel Aviv-Yaffo, Tel Aviv-Yaffo 6818211, Israel; danamc@mta.ac.il; 4 The Louis and Gabi Weisfeld School of Social Work, Bar-Ilan University, Ramat Gan 5290002, Israel; taubman@biu.ac.il

**Keywords:** mothers, trauma, pandemic, war, intrusive thoughts, dissociative experiences

## Abstract

Early caregiving can evoke feelings of helplessness in mothers that are potentially associated with disintegrative responses, i.e., intrusive thoughts and dissociative experiences in the context of infant care. Given the associated increase in stress and exposure to life-threatening dangers, crises such as pandemics and wars may intensify these responses, but this has not previously been tested. Objectives: This cross-sectional study aimed to (1) examine maternal disintegrative responses across three contexts—a high-intensity phase of the COVID-19 pandemic, a subsequent low-intensity pandemic period, and the Israel–Hamas war—and (2) compare the two crisis periods in terms of trauma-related exposure, a damaged experience of childbirth and childcare, and their links to disintegrative responses. Method: This study had two parts and was conducted among Israeli mothers. Part 1 involved 1416 mothers across three groups: high-intensity pandemic (N = 637), low-intensity pandemic (N = 360), and war (N = 419). Part 2 involved a subsample from Part 1 comparing the 1056 mothers from the high-intensity pandemic and war groups. All the participants completed questionnaires assessing maternal disintegrative responses, trauma-related exposure, and a damaged experience of childbirth and childcare. The analyses included ANOVA and mediation models, controlling for maternal characteristics. Results: Intrusive thoughts and dissociative experiences were significantly higher during both the high-intensity pandemic and war periods compared to the low-intensity pandemic period. Trauma exposure indirectly predicted intrusive thoughts and dissociative experiences through a damaged experience of childbirth and childcare, particularly during war. Conclusions: The findings indicate that maternal disintegrative responses were higher during high-intensity crisis contexts, with the highest levels reported by mothers during the war. Trauma exposure and a damaged experience of childbirth and childcare were associated with more intrusive thoughts and dissociative experiences, underscoring the need for targeted support to protect maternal well-being and caregiving during crises.

## 1. Introduction

Alongside the joy of welcoming a new baby, early caregiving is a sensitive and challenging period for parents that requires both mothers and fathers to adapt to significant physical, emotional, and familial changes (Borelli et al., 2020; Tambelli et al., 2025). For mothers, this period can be especially intense: as well as experiencing pregnancy and childbirth, in many cultures, mothers often take on the primary caregiving role for their infants. Although the early caregiving period is undoubtedly challenging for all caregivers, a combination of biological factors, caregiving responsibilities, and increased workload places mothers at uniquely elevated risk of significant increases in mental health problems, particularly depression and anxiety (Goodman et al., 2016; Liu et al., 2022).

In addition to the heightened risk of psychiatric disorders, the high demands associated with this period may evoke feelings of ambivalence and helplessness in mothers (Huth-Bocks et al., 2016; Solomon & George, 2011). This state can potentially lead to what is described in the literature as maternal disintegrative responses: psychological episodes whereby the mother’s caregiving experience is disrupted by distressing or fragmented thoughts, feelings, or perceptions, leading to a sense of detachment or an overwhelming sense of fear (Chasson & Taubman–Ben-Ari, 2023). These responses reflect a deviation from an integrated and attuned caregiving state and primarily encompass two core phenomena: intrusive thoughts and dissociative experiences (Chasson & Taubman–Ben-Ari, 2023; Chasson et al., 2023). Intrusive thoughts can include uncontrolled ideas and images with unwanted content, which during the postpartum period often involve aggressive reflections and imagery related to potential harm to the infant, either through external accident or the mother’s actions (Brok et al., 2017; Collardeau et al., 2024; Fairbrother & Woody, 2008). Dissociative experiences involve transient or recurring episodes of disconnection or discontinuity in the integration of consciousness, memory, identity, emotion, and perception of body representation (American Psychiatric Association, 2013). In the postpartum period, these experiences may manifest as a mother’s transient feelings of alienation or detachment from her infant, herself, her maternal role, or her interactions with her infant (Chasson et al., 2023; Solomon & George, 2011).

Whereas studies point to various personal factors that may influence the extent to which women experience disintegrative responses—such as being a new mother, attachment insecurity, and experiencing childhood trauma (Chasson & Taubman–Ben-Ari, 2022, 2023), as well as empathy-related distress (Chasson et al., 2024)—less is known regarding the contextual factors that may confer greater risk of disintegrative responses, particularly environmental stressors such as national or international crises. Given that the birth of a new baby triggers mental health challenges even under normal circumstances (Shaw et al., 2006), the risk of adverse mental health outcomes may be intensified during periods of instability or crisis (Moghassemi et al., 2024; Tees et al., 2010).

This heightened risk of adverse mental health outcomes was evident during the COVID-19 pandemic, which began in China in December 2019 and rapidly spread worldwide. The pandemic brought prolonged uncertainty, health concerns, social isolation, and financial hardship (Altig et al., 2020; Brülhart et al., 2021; Lakhan et al., 2020; Necho et al., 2021). Mothers in the postpartum period were particularly vulnerable during this time (Anderson et al., 2022; Kotlar et al., 2021; Racine et al., 2021). This vulnerability was exacerbated by the uncertainty of the COVID-19 situation combined with strict pandemic-related guidelines, such as wearing a mask during labor, restrictions on support figures in the delivery room, and in some cases, even the isolation of the mother from her newborn (Babu et al., 2022; Mayopoulos et al., 2021b). These factors contributed to lower birth satisfaction (Preis et al., 2022) and higher rates of traumatic childbirth experiences and childbirth-related posttraumatic stress disorder (PTSD) in women (Babu et al., 2022; Mayopoulos et al., 2021b). Furthermore, lockdowns and social distancing limited essential support systems, compounding the emotional strain on postpartum mothers already facing health and financial concerns (Terada et al., 2021; White et al., 2023; Zhou et al., 2021). As a result, many mothers felt their childbirth experience and ability to care for their newborns were significantly compromised (Mayopoulos et al., 2021a).

Prior studies have demonstrated an increase in reported postpartum-related psychopathology among mothers during the COVID-19 period compared to routine times (e.g., Layton et al., 2021; Shuman et al., 2022; Zhang et al., 2023). However, no studies have examined maternal disintegrative responses during this period. Understanding disintegrative responses during the pandemic could show how these challenges specifically impacted the caregiving experience and also provide insight into broader general mental health and stress patterns.

Another crisis that may impact the early caregiving experience is war. In addition to managing their own emotional reactions to war, mothers and other caregivers who face war-related trauma may experience additional challenges related to their caregiving role—specifically, they may experience feelings of helplessness and frustration due to their perceived inability to protect their children (Kaitz et al., 2009; Mijalevich-Soker & Taubman–Ben-Ari, 2025a; Osofsky, 1995). Research worldwide shows that mothers of infants exposed to war and conflict often face significant emotional distress and PTSD, which can profoundly impact their caregiving (Dozio et al., 2020; Feldman & Vengrober, 2011; Lahti et al., 2019; Qouta et al., 2021). This distress may reduce positive responsiveness and increase negative reactions to the infant (Lahti et al., 2019; Qouta et al., 2021; van Ee et al., 2016), disrupting the natural, nurturing bond (Igreja, 2003). Consequently, mothers may struggle to be emotionally and physically present, which can lead to heightened arousal during interactions and difficulty maintaining a calm and supportive environment (Beebe et al., 2020, 2023; Dozio et al., 2020; Igreja, 2003; Levavi et al., 2024).

The Israel–Hamas War began on 7 October 2023, following a massive terrorist attack by Hamas against Israel. This brutal assault led to the immediate death of approximately 1200 people and the abduction of over 250 hostages, including children and the elderly, who were taken to Gaza. The attack, which was one of the deadliest in recent history, sparked a fierce and ongoing war with severe consequences for civilians in both Israel and Gaza, including widespread casualties, injuries, and displacement. The widespread violence and mass casualties caused profound trauma across large segments of the population, including both Jewish and Arab people residing in Israel (Levi-Belz et al., 2024; Palgi et al., 2024; Shrira & Palgi, 2024). Furthermore, the extensive indirect exposure to the massacre, which was amplified by the viral spread of filmed atrocities on social media, exacted a heavy psychological toll (Dahan et al., 2024; Holman et al., 2024).

Studies conducted on the impact of the Israel–Hamas war on Israeli postpartum women are scarce. Research on Israeli women suggests that the pervasive fear and uncertainty overshadowed the usual joy of pregnancy and childbirth, making it a complex and emotionally charged experience (Helpman et al., 2024; Ring et al., 2024). In addition, findings indicate that during the war, mothers reported higher levels of depression and anxiety compared to women who gave birth under normal circumstances (Klapper-Goldstein et al., 2024). Pregnant women also reported better mental health during the COVID-19 pandemic compared to the war (Mijalevich-Soker & Taubman–Ben-Ari, 2025b).

Notably, while both war and pandemic contexts are characterized by heightened stress and disruption to maternal functioning, they differ in the immediacy and nature of the threat. War often evokes a more acute and externally driven sense of danger that is characterized by more immediate danger and may elicit more intrusive and alarming maternal thoughts (Fairbrother & Woody, 2008; Osofsky, 1995). In contrast, dissociative experiences are commonly understood as defensive responses to overwhelming stress or trauma (Morgan et al., 2001); hence, they may be triggered in both war and pandemic contexts due to their prolonged, destabilizing nature and emotional toll on caregiving.

### 1.1. Current Investigation

This cross-sectional study is grounded in the understanding that external crises, such as pandemics and wars, challenge the stability and security of daily life and impose significant stress on individuals, including on their early caregiving experiences. We propose that disintegrative responses in caregiving—namely intrusive thoughts and dissociative experiences—are heightened during periods of elevated societal stress compared to lower-stress periods. Additionally, we suggest that these responses may manifest differently across various types of crises due to their unique stressors. This study was therefore conducted in two parts to examine how disintegrative responses vary across a societal stress continuum.

Part 1 examined how these responses manifested during three distinct societal stress phases: a high-intensity phase of the COVID-19 pandemic (July 2020, during the second wave and pre-vaccination period), a low-intensity pandemic period (March 2022, following widespread vaccination and the lifting of restrictions), and the Israel–Hamas war (March 2024). Using three samples of Israeli postpartum women with infants aged 1 to 4 months, we compared maternal disintegrative responses (intrusion and dissociation) across these periods.

**Figure 1:**
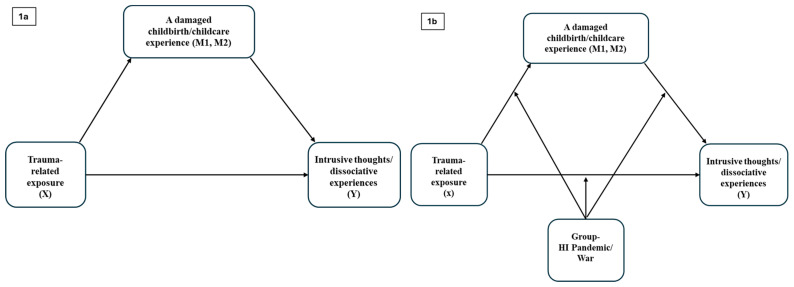
Schematic mediation (**1a**) and moderated mediation (**1b**) models for intrusive thoughts and dissociative experiences. Note. HI Pandemic = high-intensity pandemic.

Part 2 provided a more focused comparison of the two higher-intensity crisis contexts (the peak of the pandemic [high-intensity pandemic] and the war), using the same samples and crisis groups as in Part 1 (i.e., excluding the low-intensity pandemic group), to better consider the characteristics of the crisis events. In Part 2, we first assessed the differences in the level of crisis exposure and the extent to which women felt the crisis negatively impacted their childbirth experience and infant caregiving. We then tested whether a damaged experience of childbirth and childcare mediated the relationship between crisis exposure and the outcomes of intrusive thoughts and dissociative experiences in caregiving. We further explored a moderated mediation model to examine whether the type of crisis (high-intensity pandemic or war) moderated this mediation effect (see the schematic model in Figure 1a,b).

Given the distinct nature of each crisis and the unique stressors associated with both the pandemic and war, we formulated the following hypotheses for each part of the study.

### 1.2. Part 1

**H1.**  *Intrusive thoughts will be more prevalent during both the high-intensity pandemic and the war periods compared to the low-intensity pandemic period, and they will be higher during the war than during the high-intensity pandemic, reflecting the immediacy of life-threatening dangers associated with war.*

**H2.**  *Dissociative experiences will be more frequent during both the high-intensity pandemic and war periods compared to the low-intensity pandemic period. No significant differences are expected between the high-intensity pandemic and war periods, as mothers may struggle to remain present with their infant amidst the heightened stress and tension of each crisis.*

### 1.3. Part 2

**H3.**  *A damaged experience of childbirth and childcare will mediate the relationship between crisis exposure and the outcomes of intrusive thoughts and dissociative experiences.*

Finally, given the limited knowledge in the existing literature, the following research questions (RQs) were formulated:

RQ1. Are there differences in crisis exposure levels and a damaged experience of childbirth and childcare between the high-intensity pandemic and war groups?
RQ2. Does the type of crisis (high-intensity pandemic or war) moderate the mediation effect between crisis exposure and maternal disintegrative responses?

## 2. Methods

### 2.1. Procedure

#### 2.1.1. Study Design

Aiming to examine maternal mental health after childbirth, three convenience samples of Israeli mothers with infants up to 4 months old were recruited in three phases: (1) high-intensity pandemic—in July 2020, during the peak of the second wave of the COVID-19 outbreak in Israel and before vaccine availability; (2) low-intensity pandemic—in March 2022, following widespread vaccination and a significant reduction in public restrictions, although the broader pandemic context persisted; and (3) war—in March 2024, five months after the Hamas attack on Israel on 7 October and during the ongoing war in Gaza and northern Israel.

#### 2.1.2. Setting

In all cases, a request to participate in the study was posted on social media groups for mothers. This was directed specifically at mothers whose children were no more than 16 weeks old. Thus, the women who participated in the first and third phases gave birth within the same time frame after the onset of the traumatic event (i.e., the outbreak of the pandemic, the outbreak of the war). A link to an electronic version of the questionnaire was provided. The inclusion criteria were being 18 years of age or older, up to 16 weeks after childbirth, and Hebrew-speaking. To encourage participation and as a token of appreciation, the respondents who expressed interest were entered into a raffle for gift vouchers.

#### 2.1.3. Ethical Considerations

The data were collected in accordance with APA ethical standards through three stand-alone studies, each of which received ethical approval from the Ethics Committee of the School of Social Work at Bar-Ilan University: sample 1 (high-intensity pandemic)—IRB protocol 061903/2, approved on 8 July 2020; sample 2 (low-intensity pandemic)—IRB protocol 012216, approved on 7 February 2022; sample 3 (war)—IRB protocol 032404, approved on 13 March 2024.

The opening page of the questionnaire for each study assured the mothers of the anonymity and confidentiality of their responses and explained that completing the questionnaire constituted their consent to participate in the study. It also explained that the mothers could withdraw their participation at any stage. Additionally, the participants were informed that if they experienced any distress during or after completing the questionnaire, they could contact the researchers via phone or email. The researchers’ contact details were provided, along with contact information for counseling services.

### 2.2. Participants

The final sample consisted of 1416 mothers: 637 in the high-intensity pandemic group, 360 in the low-intensity pandemic group, and 419 in the war group. In all three samples, eligibility criteria for participation included being over the age of 18, up to 16 weeks postpartum, and able to complete the questionnaire in Hebrew. Table 1 presents the descriptive statistics for the entire sample and the individual groups, and includes the results of difference tests comparing the groups. The results indicated that the mothers in the war group were somewhat older and had slightly higher education levels than those in the other two groups. Conversely, the mothers in the low-intensity pandemic group reported higher economic status compared to the other groups. Finally, the high-intensity pandemic group included a higher proportion of multiparous women compared to the other groups. Maternal age, economic status, and parity were controlled for in the analyses.

### 2.3. Measures

#### 2.3.1. Maternal Disintegrative Responses Scale

The Maternal Disintegrative Responses Scale (MDRS; Chasson & Taubman–Ben-Ari, 2023) was used to assess the disintegrative responses of mothers. The scale consists of two dimensions: intrusive thoughts (four items), which reflect the mother’s experience of unwanted and uncontrolled thoughts when with or caring for the baby (e.g., “When I’m holding the baby, the uncontrollable thought that I’m going to drop him/her flits through my mind”), and dissociative experiences (4 items), which reflect the mother’s feelings of detachment and alienation from herself, her baby, or reality when with or caring for the infant (e.g., “When I’m with the baby or caring for him/her, I feel as if I’m not really there but only watching from a distance”). The participants were asked to rate how often they had the experience described in each item in the past month, from 0 (never) to 4 (very often). A score was calculated for each dimension by averaging the participant’s responses to the relevant items, with higher scores indicating higher levels of intrusive thoughts or dissociative experiences. In the current study, Cronbach’s alpha was acceptable for all the groups: intrusive thoughts (low-intensity pandemic 0.83, high-intensity pandemic 0.82, war 0.83), dissociative experiences (low-intensity pandemic 0.75, high-intensity pandemic 0.78, war 0.78).

#### 2.3.2. Trauma-Related Exposure

To assess exposure levels in Part 2 of the study, the participants in each group answered specific questions developed to evaluate exposure to two major events: the high-intensity phase of the COVID-19 pandemic (five months after its outbreak) and the events of 7 October 2023, along with the subsequent Israel–Hamas war (five months after 7 October). For the COVID-19 pandemic high-intensity phase, participants were asked: (1) Have you contracted the coronavirus? (2) Do you know someone who has contracted the coronavirus? and (3) Have you or anyone in your household been in quarantine due to the coronavirus? These questions were developed by Taubman-Ben-Ari et al. (2020). Similarly, for the 7 October events and the subsequent war, the participants were asked (1) Did you personally witness the massacre on 7 October or the war that followed? (2) Were people close to you (family members or friends) murdered in the massacre on 7 October or in the war that followed? and (3) Do you know someone who was injured, murdered, or killed in the events of 7 October or the war that followed? These questions were developed to align with the structure used for assessing pandemic exposure (Ring et al., 2024). For each group, the number of affirmative answers was counted, with a higher count indicating a greater level of exposure (see Table 2 for the question distribution).

#### 2.3.3. Damaged Experiences of Childbirth and Childcare

We developed two single-item measures (one for childbirth and one for childcare) specifically designed to assess in Part 2 of the study the perceived impact of the high-intensity phase of the COVID-19 pandemic and the Israel–Hamas war on maternal experiences. The two items were identical for both crises, with the wording tailored to reference the specific traumatic event relevant to each context. The items were (1) To what extent do you feel that your childbirth experience was damaged by the pandemic/war? (childbirth measure) and (2) To what extent do you feel that your experience of raising your infant was damaged by the pandemic/war? (childcare measure). The responses for each measure were recorded on a 5-point Likert scale, ranging from 1 (very little) to 5 (very much) (see Table 2). As these measures were developed for the current study and each consisted of a single item intended to capture mothers’ subjective perceptions of disruption to core aspects of the early postpartum experience related to adverse events specific to this study, they were not based on existing validated measures.

#### 2.3.4. Sociodemographic Questionnaire

Background sociodemographic information was collected for both parts of the study. This included the mother’s age (continuous), education (1 = elementary, 2 = high school, 3 = post-high school, 4 = academic), economic status (1 = below average, 2 = average, 3 = above average; self-rated relative to the average household income in Israel, which was provided as a reference), and parity (0 = primiparous [new mothers], 1 = multiparous [experienced mothers]).

### 2.4. Data Analysis

Prior to the primary analyses, the data were cleaned and checked for accuracy, and descriptive statistics for all the study variables were calculated using SPSS 24. In Part 1 of the study, two univariate ANOVAs were conducted in R to examine the differences in intrusive thoughts and dissociative experiences across the three groups: low-intensity pandemic, high-intensity pandemic, and war. Maternal age, economic status, and parity were included as covariates in these models. Any significant main effects were examined using post hoc Tukey tests to identify the specific group differences. In Part 2, the descriptive statistics were first examined for trauma exposure and a damaged experience of childbirth and childcare, and then for group differences between the high-intensity pandemic and war groups. Bivariate correlations were then computed to explore the associations among the demographics and study variables.

Following this, two separate mediation models were tested—one for intrusive thoughts and another for dissociative experiences—using Model 4 in the PROCESS macro (Hayes, 2017), which estimates indirect effects in simple mediation. We then applied two moderated mediation models using Model 59 in PROCESS, which allows for testing whether the mediation effect varies by the levels of a moderator and which in this study was the group (high-intensity pandemic or war). All the models were conducted using the PROCESS package in R.

After confirming that the data were missing at random using Little’s MCAR test (χ^2^(3) = 4.32, *p* = 0.82)—which was performed on all variables with missing values that were included in the main analyses (the outcome, mediator, exposure, and covariate variables)—we performed data imputation using the MICE (multivariate imputation by chained equations) method, implemented via the mice package in R (van Buuren & Groothuis-Oudshoorn, 2011). The criterion for a significant indirect effect was based on the 95% confidence interval, which was derived from bootstrap resampling (*n* = 10,000), where 95% of estimated indirect effects differed from zero (Hayes, 2017). To address potential sources of bias, maternal age, education, economic status, and parity (primiparous/multiparous) were included as covariates in all analyses to control for their potential effects on the results.

## 3. Results

### 3.1. Part 1: Differences in Disintegrative Responses as a Function of Context

#### 3.1.1. Bivariate Correlations Between the Demographic Variables and Disintegrative Responses

As shown in Table 3, the Pearson correlations revealed context-dependent associations across the low-intensity pandemic, high-intensity pandemic, and war groups. In the low-intensity pandemic group, maternal age was not significantly correlated with intrusive thoughts or dissociative experiences. However, in the high-intensity pandemic group, younger maternal age and lower economic status were significantly associated with more intrusive thoughts and dissociative experiences. In the war group, younger maternal age and lower education were significantly linked to higher intrusive thoughts, while no significant associations were found with dissociative experiences. Parity showed significant associations across all groups, with primiparous mothers reporting more intrusive thoughts and dissociative experiences. Intrusive thoughts and dissociative experiences were significantly interrelated in all groups.

#### 3.1.2. Differences in Disintegrative Responses by Period Group

**Figure 2:**
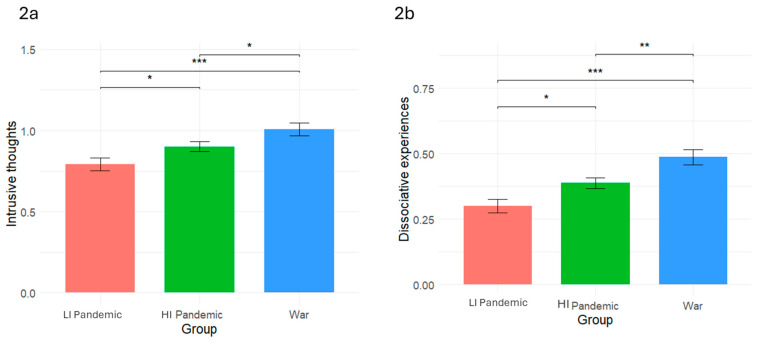
Mean differences in intrusive thoughts (**2a**) and dissociative experiences (**2b**) across crisis contexts. Note. LI Pandemic = low-intensity pandemic; HI Pandemic = high-intensity pandemic. * *p* < 0.05, ** *p* < 0.01, *** *p* < 0.001.

To test H1, we examined the differences in intrusive thoughts and dissociative experiences among the three groups. As shown in Table 1 and Figure 2a,b, there were significant differences between the groups in intrusive thoughts (*F* = 7.389, *p* < 0.001; *η*^2^ = 0.018) and dissociative experiences (*F* = 11.564, *p* < 0.001; *η*^2^ = 0.016). In line with H1, compared to the low-intensity pandemic, intrusive thoughts were significantly higher during the high-intensity pandemic (*p* = 0.035) and the war (*p* < 0.001). Additionally, consistently with H1, intrusive thoughts were significantly higher during the war than during the high-intensity pandemic (*p* = 0.030). Similarly, in line with H2, compared to the low-intensity pandemic, dissociative experiences were significantly higher during the high-intensity pandemic (*p* = 0.015) and the war (*p* < 0.001). However, contrary to H2, dissociative experiences were significantly higher during the war compared to the high-intensity pandemic (*p* = 0.004).

### 3.2. Part 2: Examining Trauma Exposure, Damaged Experience of Childbirth and Childcare, and Disintegrative Responses in the High-Intensity Pandemic and War Contexts

#### 3.2.1. Differences in Trauma Exposure and Damaged Experience of Childbirth and Childcare Between the High-Intensity Pandemic and War Groups

The means and standard deviations for trauma exposure and damaged experience of childbirth and childcare by crisis period, along with the *t*-test results, are presented in Table 2. In response to RQ1, mothers reported a significantly greater damaged childbirth experience during the high-intensity pandemic than during the war. However, no significant differences were found between the groups regarding trauma-related exposure or damaged childcare experience.

#### 3.2.2. Bivariate Correlations Between Trauma Exposure, Damaged Experience of Childbirth and Childcare, and Disintegrative Responses

As shown in Table 3, in the high-intensity pandemic group, greater damage to the childbirth and childcare experience was significantly associated with more intrusive thoughts and dissociative experiences, while trauma-related exposure was not associated with such thoughts. In the war group, both trauma-related exposure and damaged childbirth experience were significantly associated with more intrusive thoughts and dissociative experiences.

#### 3.2.3. Mediation Models

As presented in Table 4, the results of the mediation analyses revealed a direct association between trauma exposure and intrusive thoughts. However, contrary to H3, no significant indirect effects were found between trauma exposure and intrusive thoughts through a damaged experience of childbirth and childcare. Additionally, no significant direct or indirect effects were found between trauma exposure and dissociative experiences through a damaged experience of childbirth and childcare.

#### 3.2.4. Moderated Mediation Models

**Figure 3:**
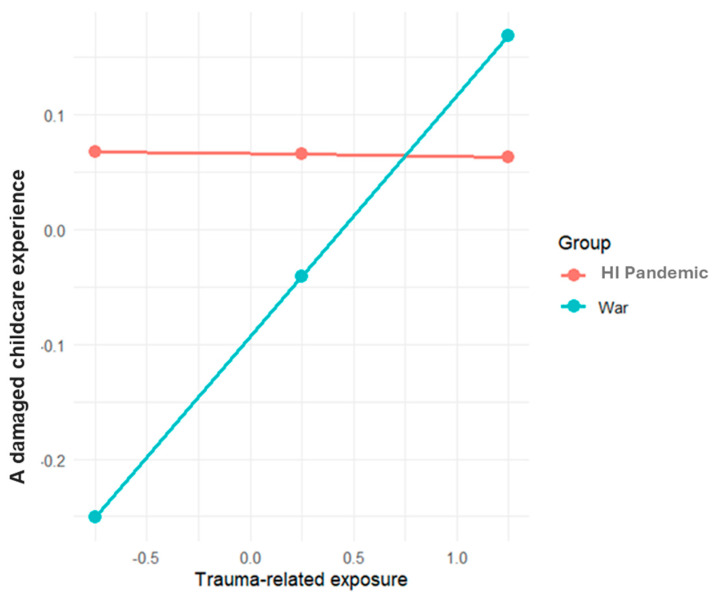
Interaction between trauma-related exposure and period group on a damaged childcare experience. Note. HI Pandemic = high-intensity pandemic. Values reflect mean-centered variables.

The results of the model testing the moderated mediation effect of trauma-related exposure in predicting intrusive thoughts by group (RQ2) revealed that the interaction between exposure to trauma and crisis group in predicting a damaged childcare experience was significant (see Table 5 and Figure 3). Thus, the positive association between mothers’ exposure to trauma and a damaged childcare experience was significant during the war (B = 0.2096, CI: 0.0552, 0.3640), but not during the high-intensity pandemic (*B* = −0.002, CI: −0.432, 0.303). Two other associations were only found to be significant for the war group: the direct association between mothers’ trauma exposure and intrusive thoughts, and the indirect effect of exposure to trauma and intrusive thoughts via a damaged childcare experience.

**Figure 4:**
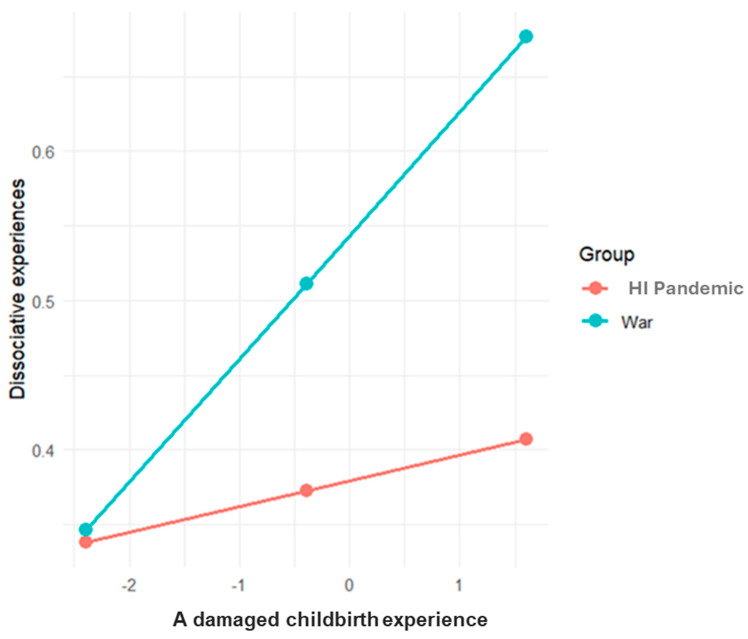
Interaction between a damaged childbirth experience and period group on dissociative experiences. Note. HI Pandemic = high-intensity pandemic. Values reflect mean-centered variables.

The results of the research model testing the moderated mediation effect for dissociative experiences by group (RQ2) are given in Table 6. As can be seen from the table, the interaction between a damaged childbirth experience and the crisis group for predicting dissociative experiences was significant. This interaction, which is shown in Figure 4, revealed that the positive association between mothers’ childbirth damage experience and dissociative experiences was significant during the war (*B* = 0.082, CI: 0.037, 0.128), but not during the high-intensity pandemic (*B* = −0.017, CI: −0.008, 0.043). The direct effect of trauma exposure on dissociative experiences was insignificant among the two groups. However, there was an indirect association between trauma exposure and dissociative experiences via a damaged childbirth experience for the war group, but not for the high-intensity pandemic group.

## 4. Discussion

This study sought to expand current knowledge on the consequences of giving birth and raising an infant during a period of crisis, specifically regarding the extent of maternal disintegrative responses, i.e., intrusive thoughts and dissociative experiences in the context of infant care.

The findings from Part 1 of this study support our hypothesis that mothers experience heightened levels of intrusive thoughts and dissociative experiences during periods of elevated societal stress—such as the high-intensity COVID-19 pandemic period and the Israel–Hamas war—compared to periods of reduced societal stress (i.e., the low-intensity COVID-19 pandemic period). These results suggest that external stressors may intensify maternal psychological symptoms, potentially making early caregiving even more emotionally demanding. Beyond individual and familial characteristics, one of the most important conditions for a positive caregiving experience is a stable and predictable environment (Appleyard & Osofsky, 2003). When this is disrupted, as happens during crises such as a pandemic or war, the caregiving experience may be destabilized (Igreja, 2003; Shuman et al., 2022).

As predicted, intrusive thoughts were significantly higher in both the high-intensity pandemic and war groups compared to the low-intensity pandemic group, with the highest levels reported by the war group. In this study, the war context, which is characterized by heightened levels of violence, hostility, and physical harm, may have contributed to the increase in intrusive thoughts, which often focus on themes of harm and aggression (Fairbrother & Woody, 2008). The traumatic scenes and characteristic violence of wartime may have overwhelmed the mothers’ consciousness, triggering intense feelings of fear and terror that were projected onto their thoughts during infant caregiving. In contrast, while the high-intensity phase of the COVID-19 pandemic posed a serious health threat, it lacked the overt violence and immediate physical danger seen in war, resulting in a different kind of psychological impact. The pandemic’s emphasis on isolation and social distancing likely fostered feelings of loneliness and separation (Terada et al., 2021; White et al., 2023; Zhou et al., 2021), rather than the heightened arousal and vigilance typically associated with war. Consequently, although high-intensity pandemic-related experiences and distress elicited more intrusive thoughts compared to the low-intensity pandemic situation, they did not provoke thoughts of direct harm or aggression to the same degree as wartime experiences.

Further reinforcement of this explanation can be seen in the moderated mediation model, which showed that the direct effect between trauma exposure and intrusive thoughts was significant only in the war group. This pattern—whereby the direct effect of trauma exposure on intrusive thoughts emerged only in the war group—aligns with prior research highlighting the intensified psychological impact of acute and hostile environments such as war (e.g., Levi-Belz et al., 2024; Shrira & Palgi, 2024). In contrast, the high-intensity pandemic group was associated with a different type of psychological response—one rooted in prolonged uncertainty and isolation (Chasson et al., 2022a; Kotlar et al., 2021)—which did not show the same direct effect between trauma exposure and intrusive thoughts as observed in the war group.

Notably, the indirect effect between trauma exposure and intrusive thoughts was also found to be significant only among mothers in the war group. This effect was explained by the interaction between the group and a damaged childcare experience, which was significant for the war group, but not for the pandemic group. These findings suggest that infant caregiving is more adversely affected among mothers exposed to war, and are consistent with previous literature linking war-related stress to disruptions in caregiving behaviors (e.g., Chasson et al., 2025; Lahti et al., 2019; van Ee et al., 2016).

Regarding dissociative experiences, the war group showed significantly higher levels compared to both the high-intensity pandemic and low-intensity pandemic groups, although mothers in the high-intensity pandemic group reported higher levels than those in the low-intensity group. This pattern suggests that caregiving under heightened societal stress—whether due to a pandemic or war—makes mothers more vulnerable to feelings of detachment and alienation, from both their infant and the caregiving role. One possible explanation for this, supported by studies conducted during the COVID-19 pandemic, is that mothers are often able to establish a sense of safety within their homes (Ashby et al., 2022; Thomson et al., 2022). This may have helped the mothers in this study to stay grounded and more connected to their caregiving during COVID-19. In contrast, during the Israel–Hamas war, even the home might have felt unsafe due to the immediate threats of violent war-related action. Consistently with previous trauma research (e.g., Chasson et al., 2025; Qouta et al., 2021), the constant threat posed by war—such as missile strikes and terror attacks—may have intensified feelings of helplessness and impaired maternal emotional availability, making it harder for the mothers in this study to feel secure and fully present with their infants.

Mothers in the high-intensity pandemic group reported having a more damaged childbirth experience. This may be explained by the strict guidelines in place during the COVID-19 period, especially during the study period, when knowledge and measures to prevent and treat the virus were limited (Taubman-Ben-Ari et al., 2025). These guidelines included strict restrictions on the number of birth companions, being required to give birth while wearing a mask, and especially strict measures imposed for women who tested positive for COVID-19 during childbirth, the latter of which were associated with a more negative and even traumatic childbirth experience for many mothers (Mayopoulos et al., 2021a, 2021b).

Interestingly, however, the relationship between a damaged childbirth experience and dissociative experiences was found to be significant only among mothers in the war group. This interaction also moderated the indirect relationship between trauma exposure and dissociative experiences within this group. Whereas both the pandemic and the war had the potential to make childbirth experiences negative and traumatic (e.g., Helpman et al., 2024; Mayopoulos et al., 2021a), several differences between these contexts may help explain the different effects on dissociative experiences. First, despite the considerable uncertainty of the COVID-19 pandemic, it was a global crisis in which women across the world shared similar childbirth experiences. This shared struggle may have fostered a sense of connection and collective reality, even in the face of ambiguity (Chasson et al., 2022b). In contrast, the Israel–Hamas war was (and still is) a geographically and socially localized trauma (Ben-Kimhy et al., 2025), which may have intensified subjective experiences of isolation and alienation among affected mothers. This lack of shared experience may have made it harder for mothers to make sense of a difficult childbirth experience during such a traumatic time. Moreover, giving birth amid widespread loss may have created inner conflict for some women, as they navigated the juxtaposition of new life and pervasive death (Helpman et al., 2024). These conflicting emotions could have intensified the emotional numbness and detachment of the women in this study, contributing to more dissociative experiences.

It is important to acknowledge several limitations in this study. First, the use of a convenience sample—while pragmatic and often necessary in times of crisis—limits the generalizability of the findings and poses a potential threat to external validity. Second, the study was not experimental, so the observed effects may be influenced by historical sequencing rather than the events themselves, especially as the data were collected sequentially: first during the high-intensity pandemic, then during a low-intensity pandemic period, and finally during the Israel–Hamas war. Additionally, the research focused exclusively on Israeli mothers, which limits the generalizability to fathers and other cultural or geopolitical contexts. The cross-sectional design also restricts insight into how maternal disintegrative responses might evolve over time or can shift as crises progress. In addition, although some background factors were controlled, the study did not assess preexisting mental health conditions, past trauma, or actual childbirth complications, all of which can influence responses. Furthermore, other relevant mental health issues, such as depression, loneliness, anxiety, and experiences of discrimination, were not measured, despite their likely impact on maternal responses. There were also potential measurement issues in that the measures assessing perceived damage to childbirth and caregiving were single-item, study-specific measures developed for this study that were not formally validated. Future studies should consider developing and validating more comprehensive tools to assess these constructs. Finally, it should be noted that while examining maternal disintegrative responses offers valuable insights, it does not serve as a diagnostic tool or clinical indicator of maternal distress or posttraumatic stress.

Notwithstanding its limitations, this study has several methodological strengths. Most notably, it enabled a comparative analysis of maternal functioning across three distinct sociopolitical contexts: a high-intensity phase of the COVID-19 pandemic, a low-intensity pandemic phase, and an active war. The inclusion of large and independent samples in each group with participants who had all given birth within a similar postpartum window enhanced the internal validity of the findings. These methodological considerations offer a strong foundation for the study’s contribution to both theory and practice. The findings highlight the important role of the environment in shaping maternal and caregiving experiences and show that a crisis environment may destabilize a mother’s sense of security, triggering more disintegrative responses during infant caregiving. Importantly, the research illustrates that different crises may impact mothers in distinct ways, specifically revealing that the Israel–Hamas war has had a significant impact on triggering both intrusive thoughts and dissociative experiences.

Major crises such as pandemics and wars heavily burden public healthcare and support systems, leaving vulnerable populations, particularly postpartum women, underserved and overlooked. To address this issue, it is essential to have support interventions and preparedness protocols. These interventions can help mothers experience childbirth and caregiving in a more positive, supported, and regulated manner, even amidst external uncertainty and violence. Practical approaches might include group-based interventions and psychoeducational tools that provide emotional support, reduce isolation, and normalize shared difficult emotions. These forms of support are not only essential for maternal mental health and the mother–infant bond but also play a crucial role in promoting optimal developmental outcomes in infants, even in times of crisis.

## Figures and Tables

**Table 1:** Descriptive statistics and group differences across maternal crisis contexts: low-intensity pandemic, high-intensity pandemic, and war.

Characteristic/ Variable	Whole Sample (*N* = 1416)	Low-Intensity Pandemic (*N* = 360)	High-Intensity Pandemic (*N* = 637)	War (*N* = 419)	Difference Test
	*M* (*SD*; Range)/% (*N*)				
Age	*M* = 31.79 (4.76; 19–47)	*M* = 30.62 (4.28; 19–45)	*M* = 31.43 (4.81; 20–46)	*M* = 33.36 (4.67; 21–47)	*F*(2, 1413) = 37.17 ***
Education					*χ^2^*(6, 1415) = 186.78, ***
Elementary	0.8% (11)	2.2% (8)	0.3% (2)	0.2% (1)	
High school	15.0% (212)	35.9% (129)	8.2% (52)	7.4% (31)	
Associate degree	8.1% (115)	7.0% (25)	9.7% (62)	6.7% (28)	
Academic degree	76.1% (1077)	54.9% (197)	81.8% (521)	85.7% (359)	
Missing	0.1% (1)	0.3% (1)			
Economic status					*χ^2^*(6, 1404) = 150.77, ***
Below average	10.3% (144)	4.6% (16)	13.6% (87)	9.8% (41)	
Average	52.1% (731)	32.2% (112)	62.3% (397)	53.0% (222)	
Above average	37.4% (529)	63.2% (220)	24.0% (153)	37.2% (156)	
Missing	0.8% (12)	3.3% (12)			
Parity					*F*(2, 1416) = 5.54 **
Primiparous	39.7% (562)	44.7% (161)	35.0% (223)	42.5% (178)	
Multiparous	60.3% (854)	55.3% (199)	65.0% (414)	57.5% (241)	
Intrusive thoughts	*M* = 0.90 (0.78; 0–4)	*M* = 0.80 (0.76; 0–4)	*M* = 0.89 (0.76; 0–4)	*M* = 1.00 (0.82; 0–4)	*F*(2, 1403) = 7.38 ***
Dissociative experiences	*M* = 0.39 (0.54; 0–4)	*M* = 0.30 (0.49; 0–4)	*M* = 0.38 (0.52; 0–4)	*M* = 0.48 (0.60; 0–4)	*F*(2, 1403) = 12.07 ***

** *p* < 0.01, *** *p* < 0.001.

**Table 2:** Frequencies of trauma-related exposure and a damaged childbirth and childcare experience in the high-intensity pandemic and war groups.

High-Intensity Pandemic			War			
Exposure to the COVID-19 Pandemic	No	Yes	Exposure to 7 October and the Israel–Hamas War	No	Yes	Difference Test
Have you contracted the coronavirus?	98.9% (*n* = 630)	1.1% (*n* = 7)	Did you personally witness the massacre on 7 October or the war that followed?	96.7% (*n* = 405)	3.3% (*n* = 14)	
Do you know someone who has contracted the coronavirus?	34.1% (*n* = 217)	65.9% (*n* = 420)	Were people close to you (family members or friends) murdered or killed in the massacre on 7 October or in the war that followed?	83.7% (*n* = 349)	16.3% (*n* = 68)	
Have you or anyone in your household been in quarantine due to the coronavirus?	42.2% (*n* = 269)	57.8% (*n* = 368)	Do you know someone or people who were injured, murdered, or killed in the events of 7 October or in the war that followed?	48.1% (*n* = 201)	51.9% (*n* = 217)	
Overall exposure *M* (*SD*)	*M* = 0.77 (0.77)			*M* = 0.71 (0.75)		*t*(1054) = 1.25 *p* > 0.05
Damaged experience of childbirth and childcare resulting from COVID-19	*M* (*SD*)	Range	Damaged experience of childbirth and childcare resulting from 7 October or the war that followed	*M* (*SD*)	Range	
To what extent do you feel that your childbirth experience was negatively affected by the COVID-19 outbreak and its consequences?	*M* = 3.76 (1.70)	1–5	To what extent do you feel that your childbirth experience was negatively affected by the war?	*M* = 2.80 (1.26)	1–5	*t*(1024.68) = 10.41 *p* < 0.001
To what extent do you feel that your caregiving experience with your baby was negatively affected by the COVID-19 outbreak and its consequences?	*M* = 2.87 (1.52)	1–5	To what extent do you feel that your caregiving experience with your baby was negatively affected by the war?	*M* = 2.75 (1.16)	1–5	*t*(1048) = 1.68 *p* > 0.05

**Table 3:** Pearson correlation coefficients among the study variables in the low-intensity pandemic, high-intensity pandemic, and war groups.

	1	2	3	4	5	6	7	8	9
1. Age									
LI Pandemic	-	0.225 ***	0.058	0.363 ***	-	-	-	0.013	−0.033
HI Pandemic	-	0.036	0.295 ***	0.373 ***	−0.165 ***	0.064	0.097	−0.128 ***	−0.133 ***
War	-	0.138 **	0.165 ***	0.264 ***	−0.118	0.068	0.000	−0.129 **	−0.117
2. Education									
LI Pandemic	-	-	0.268 **	0.143 **	-	-	-	−0.060	−0.090
HI Pandemic	-	-	0.132 ***	0.068	0.100	−0.059	−0.077	0.081	−0.063
War	-	-	0.265 ***	−0.004	−0.053	−0.053	−0.069	−0.128 **	−0.034
3. Economic status									
LI Pandemic	-	-	-	−0.045	-	-	-	−0.118	−0.058
HI Pandemic	-	-	-	0.155 ***	−0.049	0.013	0.030	−0.120 **	−0.087
War	-	-	-	0.071	−0.107	−0.075	−0.061	−0.094	−0.081
4. Parity ^d^									
LI Pandemic	-	-	-	-	-	-	-	−0.189 ***	−0.206 ***
HI Pandemic	-	-	-	-	−0.040	−0.132 ***	−0.004	−0.177 ***	−0.216 ***
War	-	-	-	-	−0.006	−0.066	−0.034	−0.206 ***	−0.160 ***
5. Trauma-related exposure									
HI Pandemic	-	-	-	-	-	−0.033	−0.022	0.090	0.007
War	-	-	-	-	-	0.100	0.129 **	0.138 **	0.100
6. Damaged childbirth experience									
HI Pandemic	-	-	-	-	-	-	0.252 ***	0.128 ***	0.087
War	-	-	-	-	-	-	0.421 ***	0.201 ***	0.206 ***
7. Damaged childcare experience									
HI Pandemic	-	-	-	-	-	-	-	0.123 **	0.055
War	-	-	-	-	-	-	-	0.251 ***	0.153 **
8. Intrusive thoughts									
LI Pandemic	-	-	-	-	-	-	-	-	0.406 ***
HI Pandemic	-	-	-	-	-	-	-	-	0.482 ***
War	-	-	-	-	-	-	-	-	0.452 ***
9. Dissociative experiences									
LI Pandemic	-	-	-	-	-	-	-	-	-
HI Pandemic	-	-	-	-	-	-	-	-	-
War	-	-	-	-	-	-	-	-	-

Note. LI Pandemic = low-intensity pandemic, HI Pandemic = high-intensity pandemic; ^d^ 0 = primiparous, 1 = multiparous. ** *p* < 0.01, *** *p* < 0.001.

**Table 4:** Unstandardized direct and indirect effects between trauma-related exposure and the two factors of maternal disintegrative responses.

Dependent Variable	Constructs	Coeff	*SE*	*t*	*p*	LLCI	ULCI	Indirect Effect	Direct Effect
Intrusive thoughts	Age	−0.007	0.005	−1.332	0.183	−0.017	0.003		
	Education	0.044	0.039	1.115	0.265	−0.033	0.122		
	Economic status	−0.074	0.036	−2.022	0.043	−0.145	−0.002		
	Parity	−0.270	0.051	−5.300	0.000	−0.370	−0.170		
	Trauma-related exposure (X)	0.087	0.031	2.797	0.005	0.026	0.087		0.08 (0.03) **
	Damaged childbirth experience (M1)	0.032	0.015	2.128	0.033	0.002	0.032	0.001 (002) ns	
	Damaged childcare experience (M2)	0.094	0.020	4.654	0.000	0.054	0.094	0.007 (0.005) ns	
Dissociative experiences	Age	−0.005	0.003	−1.443	0.149	−0.012	0.002		
	Education	−0.022	0.028	−0.789	0.429	−0.078	0.033		
	Economic status	−0.029	0.026	−1.115	0.264	−0.080	0.022		
	Parity	−0.199	0.036	−5.466	0.000	−0.270	−0.127		
	Trauma-related exposure (X)	0.0222	0.022	0.996	0.319	−0.021	0.065		0.02 (0.02) ns
	Damaged childbirth experience (M1)	0.021	0.011	1.905	0.057	−0.000	0.042	0.001 (0.001) ns	
	Damaged childcare experience (M2)	0.032	0.014	2.238	0.025	0.004	0.061	0.002 (0.002) ns	

Note. ns = not significant; ** *p* < 0.01.

**Table 5:** Unstandardized coefficients for the moderated mediation model between trauma-related exposure and intrusive thoughts.

Antecedent		Consequence				
	Damaged childbirth experience (M_1_)					
	Coefficient	*SE*	*t*	*p*	LLCI	ULCI
Trauma-related exposure	−0.034	0.079	−0.430	0.667	−0.190	0.122
Group ^1^	−1.022	0.099	−10.266	0.000	−1.217	−0.826
Exposure * group	0.204	0.126	1.613	0.107	−0.044	0.453
	Damaged childcare experience (M_2_)					
	Coefficient	*SE*	*t*	*p*	LLCI	ULCI
Trauma-related exposure	−0.002	0.063	−0.038	0.969	−0.126	0.121
Group	−0.159	0.079	−2.022	0.043	−0.314	−0.004
Exposure * group	0.212	0.100	2.110	0.035	0.014	0.409
	Intrusive thoughts (Y)					
	Coefficient	*SE*	*t*	*p*	LLCI	ULCI
Trauma-related exposure	0.071	0.039	1.809	0.070	−0.006	0.148
Damaged childbirth experience	0.041	0.018	2.233	0.025	0.005	0.077
Damaged childcare experience	0.068	0.024	2.753	0.006	0.019	0.117
Group	0.199	0.053	3.756	0.000	0.095	0.303
Exposure * roup	0.027	0.063	0.441	0.658	−0.095	0.151
Damaged childbirth experience * group	0.030	0.037	0.823	0.410	−0.042	0.103
Damaged childcare experience * group	0.058	0.043	1.347	0.178	−0.026	0.143
[Model R = 0.31; R^2^ = 0.10; MSE = 0.572, *F* [1056] = 10.582; *p* < 0.001]						
Conditional direct effect at different levels of the moderator group: trauma exposure → intrusive thoughts						
Group	Effect	*SE*	*t*	*p*	LLCI	ULCI
High-intensity pandemic	0.071	0.039	1.809	0.070	−0.006	0.148
War	0.099	0.049	2.002	0.045	0.002	0.196
Conditional indirect effect at different levels of the moderator group: trauma exposure → childbirth damage → intrusive thoughts						
Group	Effect		*SE*	LLCI	ULCI	
High-intensity pandemic	−0.001		0.003	−0.009	0.006	
War	0.012		0.009	−0.001	0.033	
Conditional indirect effect at different levels of the moderator group: trauma exposure → childcare damage → intrusive thoughts						
Group	Effect		*SE*	LLCI	ULCI	
High-intensity pandemic	−0.000		0.004	−0.009	0.009	
War	0.026		0.013	0.004	0.057	

Note. Group ^1^ = 0 = high-intensity pandemic, 1 = war. For the full model including the demographic covariates, see Supplemental Table S1.

**Table 6:** Unstandardized coefficients for the moderated mediation model between trauma-related exposure and dissociative experiences.

Antecedent		Consequence				
	Damaged childbirth experience (M_1_)					
	Coefficient	SE	*t*	*p*	LLCI	ULCI
Trauma-related exposure	−0.034	0.079	−0.430	0.667	−0.190	0.122
Group ^1^	−1.022	0.099	−10.266	0.000	−1.217	−0.826
Exposure * group	0.204	0.126	1.613	0.107	−0.044	0.453
	Damaged childcare experience (M_2_)					
	Coefficient	SE	*t*	*p*	LLCI	ULCI
Trauma-related exposure	−0.002	0.063	−0.038	0.969	−0.126	0.121
Group	−0.159	0.079	−2.022	0.043	−0.314	−0.004
Exposure * group	0.212	0.100	2.110	0.035	0.014	0.409
	Dissociative experiences (Y)					
	Coefficient	SE	*t*	*p*	LLCI	ULCI
Trauma-related exposure	−0.001	0.028	−0.067	0.946	−0.057	0.053
Damaged childbirth experience	0.017	0.013	1.318	0.187	−0.008	0.043
Damaged childcare experience	0.021	0.017	1.183	0.236	−0.013	0.055
Group	0.164	0.037	4.352	0.000	0.090	0.238
Exposure * group	0.051	0.044	1.144	0.252	−0.036	0.139
Damaged childbirth experience * group	0.065	0.026	2.462	0.014	0.013	0.117
Damaged childcare experience * group	0.010	0.030	0.344	0.730	−0.049	0.071
[Model R = 0.28; R^2^ = 0.07; MSE = 0.280, *F* [1056] = 8.24; *p* < 0.001]						
Conditional direct effect by group: trauma exposure → dissociative experiences						
Group	Effect	SE	*t*	*p*	LLCI	ULCI
High-intensity pandemic	−0.001	0.028	−0.067	0.946	−0.057	0.053
War	0.049	0.035	1.402	0.161	−0.019	0.118
Conditional indirect effect at different levels of the moderator group: trauma exposure → childbirth damage → dissociative experiences						
Group	Effect		*SE*	LLCI	ULCI	
High-intensity pandemic	−0.001		0.001	−0.004	0.002	
War	0.014		0.008	0.000	0.034	
Conditional indirect effect at different levels of the moderator group: trauma exposure → childcare damage → dissociative experiences						
Group	Effect		*SE*	LLCI	ULCI	
High-intensity pandemic	−0.000		0.001	−0.003	0.003	
War	0.006		0.006	−0.004	0.022	

Note. Group ^1^ = 0 = high-intensity pandemic, 1 = war. For the full model including the demographic covariates, see Supplemental Table S2.

## Data Availability

The raw data supporting the conclusions of this article will be made available by the authors on request.

## Supplementary Materials

The following supporting information can be downloaded at https://www.mdpi.com/article/10.3390/bs15060717/s1. Table S1: Unstandardized coefficients for the moderated mediation model between trauma-related exposure and intrusive thoughts (including demographic covariates); Table S2: Unstandardized coefficients for the moderated mediation model between trauma-related exposure and dissociative experiences (including demographic covariates).
