# Supplementary material for: Maternal Intrusive Thoughts and Dissociative Experiences in the Context of Early Caregiving Under Varying Levels of Societal Stress
Source: Behav Sci (Basel). 2025 May 23;15(6):717. doi: 10.3390/bs15060717 (PMC12189773; doi:10.3390/bs15060717)
Supplement: Supplementary file 1 [file behavsci-15-00717-s001.zip › behavsci-3586580-supplementary.pdf]

Table S1. *Unstandardized Coefficients for the Moderated Mediation Model between Trauma-Related Exposure and Intrusive Thoughts (Including Demographic Covariates)*

	Consequent					
	A damaged childbirth experience (M_1)					
Variable	Coefficient	*SE*	*t*	*p*	LLCI	ULCI
Constant	0.538	0.432	1.243	0.213	-0.311	1.387
Trauma-related exposure	-0.034	0.079	-0.430	0.667	-0.190	0.122
Group[1]	-1.022	0.099	-10.266	0.000	-1.217	-0.826
Exposure * Group	0.204	0.126	1.613	0.107	-0.044	0.453
Maternal age	0.040	0.010	3.682	0.000	0.018	0.061
Education	-0.135	0.080	-1.690	0.091	-0.292	0.021
Economic status	-0.047	0.073	-0.648	0.516	-0.192	0.096
Parity[2]	-0.468	0.103	-4.543	0.000	-0.670	-0.266
	A damaged childcare experience (M_2)					
Variable	Coefficient	*SE*	*t*	*p*	LLCI	ULCI
Constant	0.158	0.343	0.462	0.643	-0.514	0.832
Trauma-related exposure	-0.002	0.063	-0.038	0.969	-0.126	0.121
Group	-0.159	0.079	-2.022	0.043	-0.314	-0.004
Exposure * Group	0.212	0.100	2.110	0.035	0.014	0.409
Maternal age	0.022	0.008	2.551	0.010	0.005	0.038
Education	-0.163	0.063	-2.575	0.010	-0.287	-0.038
Economic status	-0.012	0.058	-0.208	0.835	-0.126	0.102
Parity	-0.091	0.081	-1.121	0.262	-0.252	0.068
	Intrusive thoughts (Y)					
Variable	Coefficient	*SE*	*t*	*p*	LLCI	ULCI
Constant	1.705	0.214	7.952	0.000	1.284	2.126
Trauma-related exposure	0.071	0.039	1.809	0.070	-0.006	0.148
A damaged childbirth experience	0.041	0.018	2.233	0.025	0.005	0.077
A damaged childcare experience	0.068	0.024	2.753	0.006	0.019	0.117
Group	0.199	0.053	3.756	0.000	0.095	0.303
Exposure * Group	0.027	0.063	0.441	0.658	-0.095	0.151
A damaged childbirth experience* Group	0.030	0.037	0.823	0.410	-0.042	0.103
A damaged childcare experience* Group	0.058	0.043	1.347	0.178	-0.026	0.143
Maternal age	-0.011	0.005	-2.099	0.036	-0.022	-0.000
Education	0.045	0.039	1.147	0.251	-0.032	0.123
Economic status	-0.080	0.036	-2.204	0.027	-0.152	-0.008
Parity	-0.237	0.051	-4.610	0.000	-0.338	-0.136
[Model R = .31; R^2 = .10; MSE = .572, *F*[1056] = 10.582; p < .001]						
Conditional direct effect at different levels of the moderator group : Trauma exposure → Intrusive thoughts						
Group	Effect	*SE*	*t*	*p*	LLCI	ULCI
High-intensity pandemic	0.071	0.039	1.809	0.070	-0.006	0.148
War	0.099	0.049	2.002	0.045	0.002	0.196
Conditional indirect effect at different levels of the moderator group: Trauma exposure → Childbirth damage → Intrusive thoughts						
Group	Effect		*SE*	LLCI	ULCI	
High-intensity pandemic	-0.001		0.003	-0.009	0.006	
War	0.012		0.009	-0.001	0.033	
Conditional indirect effect at different levels of the moderator group: Trauma exposure → Childcare damage → Intrusive thoughts						
Group	Effect		*SE*	LLCI	ULCI	
High-intensity pandemic	-0.000		0.004	-0.009	0.009	
War	0.026		0.013	0.004	0.057	

Note. Group[1] = 0 = High-intensity pandemic, 1 = War; Parity[2] = 0 = Primiparous, 1 = Multiparous

Table S2. *Unstandardized Coefficients for the Moderated Mediation Model between* Trauma-Related Exposure *and Dissociative Experiences (Including Demographic Covariates)*

Antecedent	Consequent					
	A damaged childbirth experience (M_1)					
Variable	Coefficient	SE	*t*	*p*	LLCI	ULCI
Constant	0.538	0.432	1.243	0.213	-0.311	1.387
Trauma-related exposure	-0.034	0.079	-0.430	0.667	-0.190	0.122
Group[1]	-1.022	0.099	-10.266	0.000	-1.217	-0.826
Exposure * Group	0.204	0.126	1.613	0.107	-0.044	0.453
Maternal age	0.040	0.010	3.682	0.000	0.018	0.061
Education	-0.135	0.080	-1.690	0.091	-0.292	0.021
Economic status	-0.047	0.073	-0.648	0.516	-0.192	0.096
Parity[2]	-0.468	0.103	-4.543	0.000	-0.670	-0.266
	A damaged childcare experience (M_2)					
Variable	Coefficient	SE	*t*	*p*	LLCI	ULCI
Constant	0.158	0.343	0.462	0.643	-0.514	0.832
Trauma-related exposure	-0.002	0.063	-0.038	0.969	-0.126	0.121
Group	-0.159	0.079	-2.022	0.043	-0.314	-0.004
Exposure * Group	0.212	0.100	2.110	0.035	0.014	0.409
Maternal age	0.022	0.008	2.551	0.010	0.005	0.038
Education	-0.163	0.063	-2.575	0.010	-0.287	-0.038
Economic status	-0.012	0.058	-0.208	0.835	-0.126	0.102
Parity	-0.091	0.081	-1.121	0.262	-0.252	0.068
	Dissociative experiences (Y)					
Variable	Coefficient	SE	*t*	*p*	LLCI	ULCI
Constant	1.135	0.152	7.425	0.000	0.835	1.435
Trauma-related exposure	-0.001	0.028	-0.067	0.946	-0.057	0.053
A damaged childbirth experience	0.017	0.013	1.318	0.187	-0.008	0.043
A damaged childcare experience	0.021	0.017	1.183	0.236	-0.013	0.055
Group	0.164	0.037	4.352	0.000	0.090	0.238
Exposure * Group	0.051	0.044	1.144	0.252	-0.036	0.139
A damaged childbirth experience* Group	0.065	0.026	2.462	0.014	0.013	0.117
A damaged childcare experience* Group	0.010	0.030	0.344	0.730	-0.049	0.071
Maternal age	-0.009	0.003	-2.349	0.019	-0.016	-0.001
Education	-0.020	0.028	-0.737	0.460	-0.076	0.034
Economic status	-0.032	0.026	-1.246	0.212	-0.083	0.018
Parity	-0.175	0.036	-4.766	0.000	-0.247	-0.103
[Model R = .28; R^2 = .07; MSE = .280, $F[1056] = 8.24$; $p < .001$]						
Conditional direct effect by group: Trauma exposure → Dissociative experiences						
Group	Effect	SE	*t*	*p*	LLCI	ULCI
High-intensity pandemic	-0.001	0.028	-0.067	0.946	-0.057	0.053
War	0.049	0.035	1.402	0.161	-0.019	0.118
Conditional indirect effect at different levels of the moderator group: Trauma exposure → Childbirth damage → Dissociative experiences						
Group	Effect		*SE*	LLCI	ULCI	
High-intensity pandemic	-0.001		0.001	-0.004	0.002	
War	0.014		0.008	0.000	0.034	
Conditional indirect effect at different levels of the moderator group: Trauma exposure → Childcare damage → Dissociative experiences						
Group	Effect		*SE*	LLCI	ULCI	
High-intensity pandemic	-0.000		0.001	-0.003	0.003	
War	0.006		0.006	-0.004	0.022	

Note. Group[1] = 0 = High-intensity pandemic, 1 = War; Parity[2] = 0 = Primiparous, 1 = Multiparous
